# The Mechanisms of Effector Th Cell Responses Contribute to Treg Cell Function: New Insights into Pathogenesis and Therapy of Asthma

**DOI:** 10.3389/fimmu.2022.862866

**Published:** 2022-07-11

**Authors:** Wenjing Chen, Yuxue Cao, Yuanyuan Zhong, Jing Sun, Jingcheng Dong

**Affiliations:** ^1^ Department of Integrative Medicine, Huashan Hospital, Fudan University, Shanghai, China; ^2^ Institute of Integrative Medicine, Fudan University, Shanghai, China

**Keywords:** Th2, Th1, Th17, Th9, Tfh, immunosuppression, Treg

## Abstract

CD4 ^+^ helper T (Th) cell subsets are critically involved in the pathogenesis of asthma. Naive Th cells differentiate into different subsets under the stimulation of different sets of cytokines, and the differentiation process is dominantly driven by lineage specific transcription factors, such as T-bet (Th1), GATA3 (Th2), RORγt (Th17) and Foxp3 (Treg). The differentiation mechanisms driven by these transcription factors are mutually exclusive, resulting in functional inhibition of these Th subsets to each other, particularly prominent between effector Th cells and Treg cells, such as Th2 versus Treg cells and Th17 versus Treg cells. Being of significance in maintaining immune homeostasis, the balance between effector Th cell response and Treg cell immunosuppression provides an immunological theoretical basis for us to understand the immunopathological mechanism and develop the therapy strategies of asthma. However, recent studies have found that certain factors involved in effector Th cells response, such as cytokines and master transcription factors (IL-12 and T-bet of Th1, IL-4 and GATA3 of Th2, IL-6 and RORγt of Th17), not only contribute to immune response of effector Th cells, but also promote the development and function of Treg cells, therefore bridging the interplay between effector Th cell immune responses and Treg cell immunosuppression. Although we have an abundant knowledge concerning the role of these cytokines and transcription factors in effector Th cell responses, our understanding on their role in Treg cell development and function is scattered thus need to be summarized. This review summarized the role of these cytokines and transcription factors involved in effector Th cell responses in the development and function of Treg cells, in the hope of providing new insights of understanding the immunopathological mechanism and seeking potential therapy strategies of asthma.

## Introduction

Asthma is a common chronic respiratory inflammatory disease where Th cell subsets play a crucial role (1). Several studies on patients with asthma have indicated abnormalities in Th cell immune response, including excessive effector Th cell response and impaired Treg cell immunosuppressive function (2). On the one hand, activated effector Th cells, including Helper T type 1 (Th1), Th2, Th17 and Follicular helper T (Tfh) cells, produce a large number of inflammatory mediators including chemokines and cytokines, which interact with other immune cells or structural cells, leading to the development of asthma pathological features such as airway inflammation and airway remodeling; On the other hand, immunosuppressive regulatory T (Treg) cells show decrease in number and impairment in inhibition function, resulting in the failure of constraint and resolution of ongoing inflammation, eventually leading to chronic inflammatory disorders (2).

Naive Th cells differentiate into distinct Th cell subsets in response to ambient cytokines, dominant by lineage specific transcription factors, including T-box transcription factor ( T-bet, also known as Tbx21), GATA binding protein 3 (GATA3), retinoid-related orphan receptor-gammat (RORγt) and Forkhead box protein P3 (Foxp3), leading to Th1, Th2, Th17 and Treg differentiation, respectively (3). Importantly, the differentiation processes mediated by these transcription factors are mutually exclusive, resulting in functional antagonism among these Th cell subsets, particularly prominent between effector Th cells and Treg cells, such as Th2 versus Treg cells and Th17 versus Treg cells (3). During an immune response, effector Th cells and Treg cells generally play opposite roles with the former promoting and the latter suppressing the immune inflammation. Furthermore, inflammatory cytokines produced by effector Th cells, such as IL-4(Th2) (4, 5), and IL-17(Th17) (6, 7), have been found to prevent Treg cell development and function, while Treg cells in turn repress effector Th cell responses through a variety of mechanisms (8). In view of its important role in maintenance of immune homeostasis, the role of the counterbalance between effector Th cell response and Treg cell immunosuppression has always been an attractive topic to explore in multiple immune mediated diseases, including asthma (9, 10).

Currently, our understanding of the balance between effector Th cell response and Treg cell immunosuppression in asthma is mostly based on the concept that effector Th cells and Treg cells are inhibitory to each other, as indicated in many studies (11–13). However, the relationship between effector Th cells and Treg cells seems to be much more complex than this concept describes. A series of studies have shown that cytokines and transcription factors involved in effector Th cell responses such as IFN-γ and T-bet of Th1, IL-4 and GATA3 of Th2, IL-6 and RORγt of Th17, drive effector Th cells mediated immune response, playing proinflammatory roles; On the other hand, these cytokines and transcription factors also promote the inhibitory function of Treg cells, serving as immunoregulators (14), indicating that the mechanisms underlying effector Th cell response and Treg cell immunosuppression are not completely opposite.

**Figure 1:**
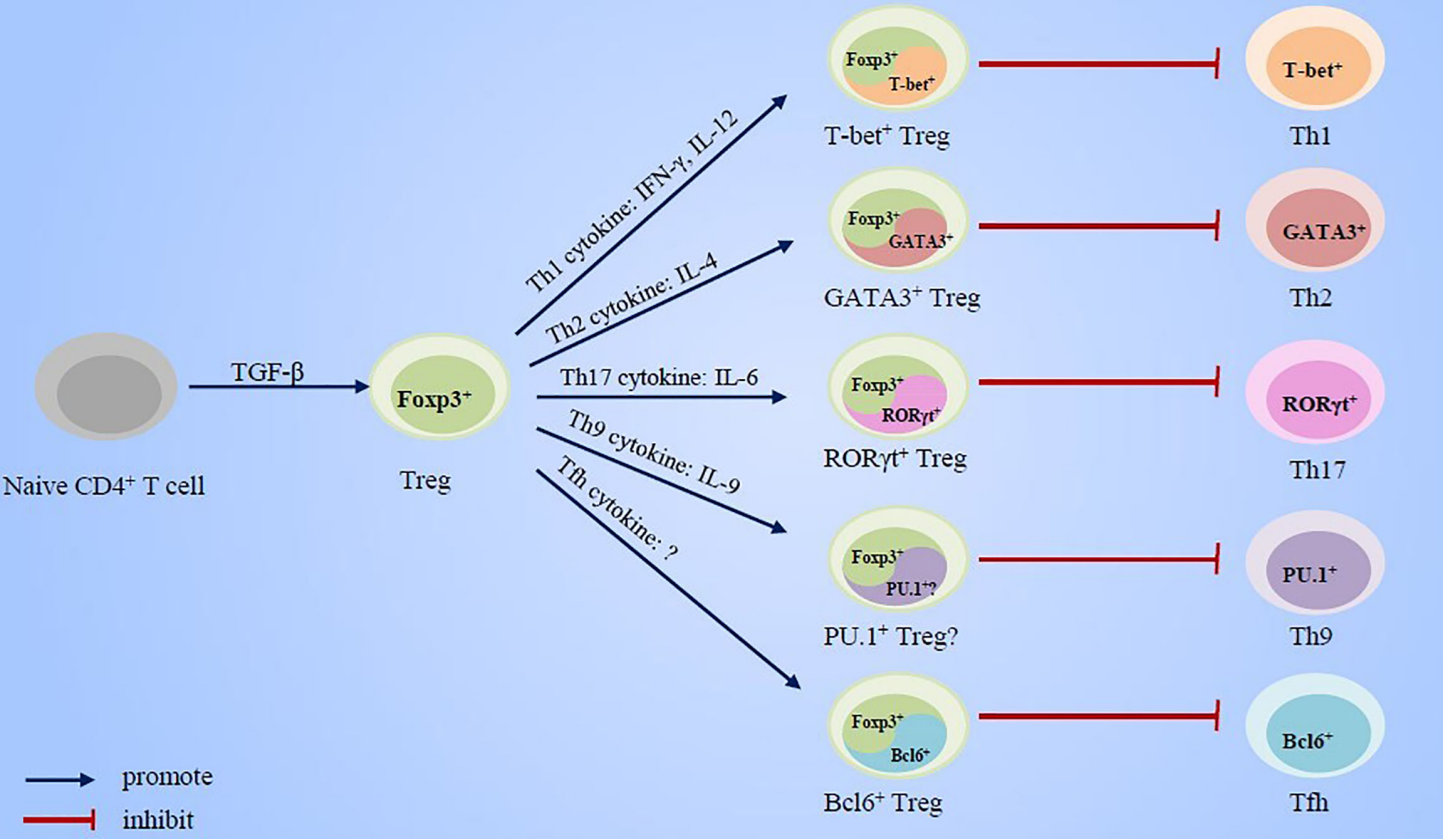
Cytokines and transcript factors of effector Th cells promote the specialization and enhancement of Treg cell function.

In this review, we summarized the research advancement concerning the essential roles of cytokines and transcription factors, which has been usually implicated in effector Th cell response, in Treg cell development and immunosuppressive function (**Figure 1**). Based on these evidences, we put forward new views on the regulation mechanisms of the counterbalance between effector Th cells and Treg cells in the pathogenesis of asthma and also briefly discuss the potential value of these research advances for asthma treatment strategies.

## The Promotions of Treg Functions by Effector Th Cell Mechanisms

Early animal studies have observed that CD25 expression by peripheral Treg cells is dependent on the presence of CD25^-^CD4^+^ T cells, suggesting the indispensability of CD25^-^CD4^+^ T cells in maintaining the stability of CD25^+^ Treg cells (15, 16). Moreover, there are compelling evidences showing that effector Th cells activation strongly boosts the expansion and suppressive activity of Treg cells (17). Although the mechanism by which effector Th cells promote the stability and inhibitory function of Treg cells has not been fully elucidated, the essential roles of cytokines and transcription factors involved in the effector Th cell responses in the development and function of Treg cells have been increasingly revealed.

### Promotions by Th2 cell mechanisms: IL-4, IRF4 and GATA3

IL-4 is a well-known cytokine that promotes Th2 differentiation, and its contribution in Th2 immune response has been widely confirmed (18). Nevertheless, IL-4 signaling was found to play a vital role in the development and function of CD25^+^ Treg cells (19). It was found that in the early development of immunosuppressive Treg cells, there was a transient high expression of IL-4 by Treg cells, which gradually declined with the acquisition of immunosuppressive phenotype by Treg cells (20), suggesting that IL-4 may participate in the development of Treg cells. During induction of antigen tolerance, blockage of IL-4 signaling with IL-4 neutralizing antibody inhibited the expansion of antigen-specific suppressive Treg cells, thus hindering the establishment of antigenic tolerance (21). In other studies, however, the loss of IL-4 signaling did not affect the development of *in vivo* Treg cell population under steady state, as the frequency of Treg cells distributed in the tissues of mice with either systemic IL-4 KO (22) or Treg cells-specific IL-4 signaling deletion (23) was similar to that of wide-type (WT) mice. Moreover, IL-4 KO Treg cells *in vitro* showed comparable proliferation as compared to WT Treg cells (22, 23). One possible explanation for the distinct results by these researches may partly be due to the different immune status investigated during the experiments, as the former studies had immunogen stimulation (21) while the latter (22, 23) did not, suggesting that the role of IL-4 on Treg cell development may to a large extent, if not fully, depend on immune status. *In vitro* studies on CD4^+^ T cells from healthy people also showed that IL-4 did not affect the Foxp3 expression by Treg cells (24)

Although the loss of IL-4 signaling did not prevent the development of Treg cell population in steady state, the inhibition function of Treg cells from IL-4 KO mice decreased compared with Treg cells from WT mice (22). Moreover, IL-4 neutralizing antibody led to gradual loss of the inhibition function of Treg cells (22) due to the downregulation of immune regulatory molecules such as IL-10, granzyme A and granzyme B (22, 25). On the contrary, recombinant IL-4 restored the impaired immunosuppressive capacity of Treg cells from IL-4 KO mice (22). These results suggested that IL-4 signaling was indispensable for maintaining Treg cells inhibitory function. Notably, it was Treg cell specific- (23, 26), but not systemic (27) or other types of immune cell specific- IL-4 signaling (28, 29) deletion that led to more serious immune inflammatory response, emphasizing the particular promotion by IL-4 signaling on Treg cell immunosuppressive function. The underlying mechanisms causing this discrepancy are worth exploring. Additionally, it was also demonstrated that IL-4 maintained the optimal regulatory function of human CD4^+^CD25^+^ Treg cell population (24)

A fact worthy of discussion was that although IL-4 was important for the inhibition function of Treg cells, neither mice with systemic IL-4 KO (22) nor mice with Treg cell specific IL-4 signaling loss (23) develop spontaneous autoimmune symptoms in a stable state, and instead they still maintained a “healthy” state similar to WT mice (22, 23). One possible explanation for the absence of spontaneous autoimmune symptoms in these mice may be the limited time window observed, as the age of the mice used in these studies was usually between 6 and 8 weeks (22, 23), when it was too early to develop detectable immune disorders, although the subtle immune damage may had already existed (23). Extending the time window of observation may contribute to the emergence of the observable spontaneous immune symptoms in these mice with IL-4 signaling defect (30). Another persuasive view was that in stable state, as the subtle immune damage may not be obvious, immune system still maintained a superficial balance, while a more inflammatory environment would more effectively expose hidden immune defects (23). Indeed, although both seem to be “healthy” in stable state, mice with systemic IL-4 signaling defect under the induction of immune response showed reduced inflammation as compared to WT mice (31), while the mice with Treg cell specific IL-4 signaling lose showed more serious inflammation (23, 26), which further supported the promotion of IL-4 on the immunosuppressive function of Treg cells. In addition, systemic IL-4 KO mice were more susceptible to parasite infection which was mainly due to the impaired Th2 immune response against parasite infection driven by IL-4 (22).

Interferon regulatory factor 4 (IRF4) is a transcription factor directly regulated by IL-4 and is crucially involved in Th2 differentiation (32). Conditional knockout of IRF4 in Treg cells led to a decrease in the number of Treg cells and obvious autoimmune symptoms in mice (33), which was different from consequences of Treg specific- or systemic IL-4 signaling loss (22, 23), indicating that the regulatory mechanism of IRF4 on Treg immunosuppressive function may be partially divergent from IL-4 signaling. IRF4 may participate in part of the inhibition programs of Treg cells, e.g. the inhibition program for Th2 response, rather than the whole immunosuppression program, because the conditional deletion of IRF4 in Treg cells led to the selective dysregulation of Th2 response in mice, but did not affect the Th1 response, while the Foxp3^-/-^ mice lacking Treg cells showed simultaneous dysregulation of Th1 and Th2 response (33). Transcriptomic analysis indicated that the deletion of IRF4 resulted in the down regulation of 20% specific genes and the up regulation of 7% specific genes in Treg cells (33). Moreover, IRF4 deficient Treg cells *in vitro* preserved the capability to inhibit Foxp3^-^CD4^+^ T cells proliferation and express inhibitory effector molecules including CTLA4, CD25 and GITR, but showed a decrease in ICOS expression (33). Inhibition of ICOS has been shown to result in impaired differentiation of Treg cells thus damaged inhibition on Th2 cell expansion (20).

GATA3 is the master transcription factor for Th2 differentiation, and IL-4 induces its expression (34).Mice with Treg cell specific GATA3 deletion develop autoimmune symptoms of splenomegaly and lymphadenopathy at the age of 16 weeks (30). Treg cells deficient in GATA3 *in vivo* were more unstable than those with normal GATA3 expression, and the expression of some signature genes such as CD25, CTLA4 and GITR decreased in these cells (30), which was similar to the consequences of IRF deletion (33). Moreover, the deletion of GATA3 also caused an increase in the expression of Th1, Th2 and Th17 effector cytokines in Treg cells, which may be due to the decreased Foxp3 expression (35). GATA3 has been shown to promote Foxp3 expression by binding to CNS2 of Foxp3 gene (30, 35). In addition, GATA3 also promoted the migration of Treg cells to inflammatory sites by promotion of homing receptor CCR8 expression (14) (35). The migration of Treg cells to inflammatory sites was reported to be crucial for Treg cells to control ongoing inflammation (36). The expression of GATA3 by Treg cells may represent the activation state of Treg cells rather than adaptation to specific immune environment, because Treg cells expressed GATA3 after TCR activation, and this process was independent on Th2 cytokines (35).

### Promotions by Th1 Cell Mechanisms: IFN-γ, T-bet and IL-12

T-bet is the master transcription factor driving Th1 differentiation. Under the condition of Th1 immune response, the number of T-bet^+^ Treg cells increased significantly, mainly due to the transformation of T-bet^-^ Treg cells into T-bet^+^ Treg cells, rather than the expansion of existing T-bet^+^ Treg cells (37, 38). Comparing the Th1 immune response systems caused by different pathogens, it was found that the transformation of T-bet^-^ Treg cells to T-bet^+^ Treg cells was the common feature of Th1 immune response and progress in parallel with the corresponding Th1 response, accompanied by Treg cells acquiring excellent migration ability as well as certain unknown characteristics favoring their controlling Th1 immune response (39). T-bet^+^ Treg cells generated *in vivo* have been proved to be a stable subset, even under the Th2 polarizing condition (38), which may be due to their epigenetic stability (40). However, some studies have also found that the expression of T-bet by Treg cells was highly dynamic in the stable state (41). Therefore, it is necessary to further clarify the factors affecting the expression of T-bet by Treg cells. The loss of T-bet expression did not affect the survival of Treg cells in stable state, but led to impaired survival and proliferation of Treg cells under Th1 inflammatory conditions (37). Moreover, more than half of T-bet^-^Treg cells transferred alone to Rag^-/-^ mice lost Foxp3 expression, while T-bet^-^Treg cells co-transferred with naive CD4^+^ T cells maintained Foxp3 expression (41).

T-bet mediated the migration of Treg cells to Th1 immune inflammatory sites by promoting the expression of CXCR3, which was crucial for Treg cells to control Th1 cells immune response (37). The deletion of T-bet had no significant effects on the expression of other chemokine receptors such as CXCR4 and CCR7 in Treg cells (37). Treg cells lacking T-bet expression still had inhibitory function (42) and Treg cell specific T-bet deletion did not affect the health of mice under 6 months of age (41), but the selective loss of T-bet in Treg cells exacerbated the existing immunopathology skewing to Th1 type inflammation (42), emphasizing the important role of T-bet in Treg cells controlling Th1 immune response. Therefore, T-bet may be involved in Treg cells inhibitory program selectively against Th1 immune response, rather than the overall inhibitory functions. It was worth noting that although mice with genetic T-bet^+^ Treg cell deficiency did not spontaneously develop immunopathology (42), timed ablation of T-bet^+^ Treg cells mediated by diphtheria toxin led to excessive Th1 immune inflammation with not affecting Th2 and Th17 responses (38). Besides, Foxp3^-/-^ mice remained healthy after receiving WT Treg cells, but developed apparent inflammatory disorders of lymphadenopathy and splenomegaly after receiving T-bet^-/-^ Treg cells (37). These results indicated that different timing of T-bet^+^ Treg cell deletion may lead to immunological consequences.

T-bet expression in Treg cells was dependent on Th1 cytokine IFN-γ, which induced the expression of T-bet in Treg cells by activating STAT1 signaling (43). During inflammatory response, IFN-γ produced by effector T cells, including CD8^+^ and CD4^+^ T cells, facilitated the expression of CXCR3 receptor and inhibitory IL-10 in Treg cells, thus promoting the functional specialization of Treg cells (43). In mice deficient in IFN-γ signaling, the generation and function of Treg cells with alloantigen and self-antigen activity were significantly constrained (44). Also, mice deficient in STAT1, a transcript factor downstream of IFN-γ, showed impaired development and function of CD25^+^ Treg cells and were more susceptible to autoimmunity (45). These results indicated that IFN-γ was essential for Treg cells to control Th1 immune response.

The mechanism by which Treg cells in response to IFN-γ selectively inhibited Th1 immune response without reprogramming to Th1 phenotype is unclear, but it may partly be due to the delayed STAT4 phosphorylation in response to IL-12 as a result of the very low expression of IL-12Rβ2 by Treg cells (43). IL-12 is a pro-inflammatory cytokine that favors the differentiation of Th1cells and induces the production of IFN-γ (46). IL-12 has been found to promote the induction of immunosuppressive Treg cells, thereby preventing allograft rejection (47). Both Foxp3^+^ CXCR3^+^ Treg cells and Foxp3^-^ CXCR3^+^ Th1 cells expressed IL-12Rβ1 subtype of IL-12 receptor, but IL-12Rβ2 expression was ~ 100 fold lower in Foxp3+CXCR3+ Treg cells (43). One reason of the very low expression of IL-12Rβ2 by Treg cells was that there was only epigenetic feature of repressive H3K27me3 histone modification on the promoter of *il12rb2* gene of Treg cells (43). Notably, although its expression was very low, IL-12Rβ2 seemed to not be redundant but instead be very important for Treg cell function. Compared with WT mice, IL-12Rβ2^-/-^ mice under stable state showed no significant difference in the absolute number of Treg cells in spleen and thymus, but showed fewer Foxp3^+^ Treg cells after TCR stimulation (48), indicating that IL-12Rβ2 may regulate the proliferation or transformation of Treg cells. Moreover, *in vitro* Treg cells from IL-12Rβ2^-/-^ mice had worse inhibitory effects on effector T cells and less proliferation after CD3 and CD28 stimulation (48). Moreover, CD25^-^CD4^+^ T cells from IL-12Rβ2^-/-^ mice showed less transformation to Treg cells under TGF-β stimulation *in vitro* (48).

### Promotions by Th17 Cell Mechanisms: IL-6, STAT3 and RORγt

Compared with Th1 and Th2 cells, Th17 cells have a closer relationship and complex interaction with Treg cells, since their differentiations are both induced by TGF-β (49) and they develop from the common progenitor cells co-expressing RORγt and Foxp3 (50). It was found that Th17 and Treg cells mutually promoted each other’s generation and expansion (51). Moreover, Th17 cells may be the most potent Th subset in the stimulation and support of expansion and phenotypic stability of Treg cells *in vivo* (52).

Studies on animal models have confirmed the important role of Th17 cells in the Treg cells stability. Compared with Rag1^-/-^ mice receiving adoptive transfer of only Treg cells, Rag1^-/-^ mice receiving co-transfer of Treg and Th17 cells showed a higher percentage of Treg cells in spleen CD4+ T cells, indicating that the presence of Th17 cells was conducive to the stability and expansion of Treg cells *in vivo* (52). TNF-TNFR2 signaling activation was one of the critical mechanisms by which Th17 cells supported the stability and expansion of Treg cells. TNFR2 plays an important role in the differentiation and inhibitory function of Treg cells (53). Although constitutively expressed TNFR2 (54, 55), Treg cells did not express TNF, the ligand of TNFR2, so they were unable to support their own stability and function through the autocrine pathway, thus needed TNF produced by other cells such as Th17 cells to activate TNFR2 (51). Among all Th cell subsets, Th17 cells have been found to had the highest TNF expression, therefore was able to most effectively promote the expansion of Treg cells and stabilize the expression of Foxp3 (52). However, whether Th17 cells promote the stability and proliferation of Treg cells in other ways remains to be further verified.

In addition to cytokines produced by Th17 cells, such as TNF, which promote the differentiation and function of Treg cells, the components of Th17 differentiation mechanism also promote the function of Treg cells. STAT3 signaling activated by IL-6 was necessary for expression of RORγt, the master transcription factor that determined Th17 differentiation (56). Animal studies have demonstrated that IL-10^-/-^ mice with IL-6 deficiency showed more severe colitis and more aggressive disease progression, and developed systemic inflammation that was absent in IL-10^-/-^ mice without IL-6 deficiency (57), indicating that IL-6 may promote IL-10 independent mechanism of Treg cells suppression function. IL-6 promoted the remarkable proliferation of human Treg cells *in vitro*, and maintained the high expression of Foxp3 and Helios, the two lineage-defining transcription factors for Treg cells, thus maintaining the stability of Treg lineage and function, regardless of the concentration of IL-2 (58). The significance of IL-6 signaling in Treg cell inhibitory function was further supported by the fact that lack of IL-6R significantly reduced Treg cells *in vitro* and *in vivo* suppressive capacity (59). IL-6 deletion also caused increased expressions of a variety of pro-inflammatory factors such as IL-4, IL-12 and TNF-α, and decreased expression of Foxp3 in mice (57), indicating that IL-6 signaling not only promoted the inhibitory function of Treg cells, but also prevented Treg cells from acquiring the pathological phenotype of generating pro-inflammatory cytokines.

STAT3 is crucial for Th17 cell differentiation (60). Interestingly, mice with Treg specific elimination of STAT3 developed Th17 type immune response at the age of 12 weeks, accompanied by anemia, weight loss, rectal prolapsed and colon thickening, the hallmarks of inflammatory bowel disease (61). Mice with Treg specific elimination of STAT3 did not develop Th1 and Th2 immune responses, suggesting that STAT3 may merely regulate the specific part of Treg cells functions, such as the part that controls Th17 immune response, without affecting the inhibitory activities of Treg cells on Th1 and Th2 responses (61). Microarray analysis showed that the deletion of STAT3 resulted in the downregulation of IL-10, Ebi3, Grmb and prf1, which were involved in the inhibitory function of Treg cells (61). Importantly, STAT3 deletion also resulted in increased expression of IL-6 and TGF-β1 by Treg cells (61), whereas TGF-β1 plus IL-6 promote transdifferentiation into Th17 cells (56). Indeed, soluble factors produced by STAT3-deficient Treg cells were able to facilitate differentiation of IL-17-producing T cells *in vitro* in the absence of exogenously supplements of IL-6 and TGF-β1 (61).

RORγt is the master transcription factor for the Th17 cells (62). It was found that 20~ 30% of Foxp3^+^ T cells in the lamina propria of the gut were RORγt^+^Foxp3^+^Treg (63), and these cells were mainly differentiated from naive conventional CD4+ cells interacting with gut microbiota (64). In addition, under inflammatory conditions, Treg cells, including thymus derived Treg cells and peripherally induced Treg cells, up regulated RORγt expression and transformed into RORγt^+^Foxp3^+^Treg (65). It has been reported that RORγt was essential for the maintenance of Foxp3 expression (66). Compared with RORγt^+^Foxp3^+^ Treg cells, RORγt^-^Foxp3^+^ Treg cells were more likely to lose Foxp3 expression (66) and had worse inhibitory ability (63). During colitis induced by adoptive transfer of CD45RB^hi^ cells into Rag1^-/-^ mice, RORγt-deficient Treg cells lost their suppressor function and acquired Th1-like effector phenotype, leading to severer colitis, suggesting that RORγt promoted Treg cells function as well as prevented Th1-like effector program in Treg cells (66).

### Promotions by Th9 Cell Mechanisms: IL-9

Th9 differentiation was induced by the simultaneous presence of IL-4 and TGF-β (67). Th9 cells produced the signature cytokine IL-9 regulating the functions of multiple kinds of immune cells, including T cells, B cells, ILCs and mast cells (68). IL-9 was also produced by other immune cells, such as ILC, Th17 and Treg cells (69–71). Although it has been widely confirmed to be associated with a variety of immune-mediated inflammatory diseases (70, 72, 73), IL-9 was also found to promote Treg cells survival and suppression function (68).

It was found that IL-9R was induced in Foxp3^+^ Treg cells during their development, and the mice with IL-9 KO showed decrease in the number of immunosuppressive Foxp3^+^ Treg cells in lung (74). Studies have shown that IL-9 promoted the survival of Treg cells by protecting Treg cells from apoptotic cell death (71, 75), possibly through the STAT3/STAT5 dependent pathway (75). The number of Treg cells in IL-9R^-/-^ mice or IL-9^-/-^ mice was equivalent to that in WT mice (69, 75), indicating that IL-9 promoted the survival of Treg cells, but may not be essential for the development of Treg cells.

Although the loss of IL-9 did not affect the general immunosuppressive function of Treg cells (76), neutralizing IL-9 reduced the expression of IL-10 by Treg cells (74) and reversed the inhibition of Treg cells on the proliferation and cytokine production of effector cells (75), which may be due to the inhibition of Foxp3 expression (71). On the contrary, recombinant IL-9 promoted the inhibition function of Treg cells *in vitro* (75). Animal studies showed that IL-9R^-/-^ mice developed more earlier and serious immune diseases after immunization with immunogens, which was verified by using IL-9 neutralizing antibody (75). In mice with allograft tolerance, neutralization of IL-9 completely reversed the prevention of graft reject by Treg cells and greatly accelerated allograft rejection (77). In the same study, IL-9 has been shown a critical factor by which activated Treg cells recruited and activated mast cells to mediate regional immune suppression (77), which was verified by other studies (76). More importantly, emerging events also demonstrated that IL-9 promoted the resolution of inflammation by regulating the expression of functional molecules of Treg cells, such as GITR and ICOS, and the capability of Treg cells from IL-9^-/-^ mice to inhibit the proliferation of CD25^-^Foxp3^-^ effector T cells was impaired (69). These results suggested that IL-9 played a critical role in the suppressive function of Treg cells.

### Promotions by Tfh Cell Mechanisms: Bcl6

Tfh cells are a distinct subset of CD4^+^ T cells specialized for helping B cells functions and are required for germinal center (GC) response (78). The GC reaction needs to be tightly regulated to avert excessive immune response (79). T follicular regulatory (Tfr) cells are a specialized subset of T regulatory cells that potently suppress both Tfh and B cells in the GC reaction (80). Tfr cells express various Tfh genes including Bcl6 (80), the transcription factor essential for Tfh differentiation (81).

Despite the lack of credible evidence that Bcl6 participated in the development of Treg cells *in vivo* (82) and regulated the inherent expression of Foxp3 (83), there was evidence that Bcl6 deletion strongly led to the instability of Treg cells and significantly promoted the loss of Foxp3 expression under Th2 immune conditions (83), indicating that Bcl6 was very important for the maintenance of Foxp3 expression and the stability of Treg cells under inflammatory conditions. In addition, Bcl6 was critical for Treg cells repression capability *via* a CXCR5 dependent way (84). Compared with WT Treg cells, both CXCR5^-/-^ and Bcl6^-/-^ Treg were significantly less efficient in suppressing the generation of germinal center B cells, affinity maturation of antibodies, and the differentiation of plasma cell although they had resemble suppression on naïve CD4^+^ T cells (84). In addition, Bcl6^-/-^ Treg cells were found to have serious defects in the ability to inhibit Th2 inflammation, though they were able to inhibit the proliferation of conventional T cells *in vitro* and prevent Th1 mediated inflammation *in vivo* as effectively as WT Treg cells (82). Compared with control mice without Bcl6^+^ Treg cell deletion, mice with Bcl6^+^ Treg cell deletion showed higher expressions of Th2 cytokines and similar expressions of Th1 and Th17 cytokines (82). These studies together showed that the inherent Bcl6 expression of Treg cells not only promoted the stability of Treg cells, but also mediated the inhibitory function of Treg cells on Tfh and Th2 responses.

## New Insights into CD4 + T Cells in Asthma Pathogenesis and Therapy

Although the imbalance of effector Th cell immune response and Treg cell immunosuppression in asthma has been broadly observed and characterized (85–88), the underlying mechanism is still unclear. It is generally believed that effector Th cell immune response and Treg cell immunosuppression are regulated by different mechanisms, resulting in different effects. However, a series of evidences abovementioned show that the effector Th cell immune response and Treg cell immunosuppression are not completely independent of and counteracting each other. Instead, they share some regulatory mechanisms, including cytokines and transcription factors, which not only promote the differentiation and immune response of effector Th cells, but also promote the development and function of Treg cells (19).

Under normal circumstances, when effector Th cell response is triggered by antigen, these shared cytokines and transcription factors not only mediated inflammation but also support the immunosuppressive function of Treg cells, so that the effector Th cell immune response can be timely and effectively regulated, which is very important to avoid excessive immune response (21). It has been commonly indicated that many cytokines shared by Th cell immune response and Treg cell immunosuppression are up-regulated in asthma, such as IL-4 (89, 90), IL-6 (91, 92), IL-9 (93, 94), etc. However, in asthma with high expression of these cytokines, the immune response of effector Th cells was activated as expected, but the immunosuppression of Treg cells did not seem to be properly activated. Therefore, the abnormal response of Treg cells to these cytokines may play a more significant role in the pathogenesis of asthma than that of activated effector Th immune response.

Reconstituting balance of Th cell immune response and Treg cell immunosuppression is an important strategy for the treatment of asthma. Based on the fact that asthma commonly is immunopathologically characterized by abnormally activated effector Th cell immune response, there is a trend in the development of new asthma drugs to inhibit the activity of key factors involved in Th cell immune response by exogenous neutralizing antibodies to achieve the purpose of preventing immune inflammation (95, 96) However, as mentioned above, the key factors involved in effector Th cell immune response also participate in Treg cell inhibitory function. Therefore, although inhibition of these key factors can effectively limit effector Th cell response, it may also lead to impairment of Treg cell inhibitory function (26). For example, it was found that administration of IL-4 antagonists in the mouse model of allergic asthma not only led to beneficial effects on immunological key parameters but also resulted in decreased ST2^+^ Treg cells (97), which has been proved to have a strong immunosuppressive effect (98). Therefore, when exploring the treatment strategies for asthma of targeted inhibition of pro-inflammatory factors such as IL-4, it is necessary to take the potential unwanted effect of impaired Treg cell function into consideration.

## Conclusion

We summarized in this review a series of evidences showing that some important mechanisms in effector Th cell immune response, including cytokines and transcription factors, not only contribute immune inflammatory response, but also play an important role in Treg cell function, thus revealing the complex relationship between effector Th cell response and Treg cell immunosuppression beyond their mutual inhibition. Because these cytokines and transcription factors have a positive regulatory effect on both effector Th cell immune response and Treg cell immunosuppression, asthma treatment strategies targeting these cytokines and transcription factors needed to be treated with caution, as they will not only affect effector Th cell immune response, but also affect Treg cell immunosuppression, resulting in unwanted consequences.

## Author Contributions

JD and JS attended discussion for the project design. WC and JS drafted the manuscript. YZ and YC revised the manuscript. All authors contributed to the article and approved the submitted version.

## Funding

This study was supported by the National Natural Science Program of China (grant No. 82174170, 81703829), Shanghai Science and Technology Commission (grant No. 18401901800, 21S21902500).

## Conflict of Interest

The authors declare that the research was conducted in the absence of any commercial or financial relationships that could be construed as a potential conflict of interest.

## Publisher’s Note

All claims expressed in this article are solely those of the authors and do not necessarily represent those of their affiliated organizations, or those of the publisher, the editors and the reviewers. Any product that may be evaluated in this article, or claim that may be made by its manufacturer, is not guaranteed or endorsed by the publisher.
